# The influence of snuff and smoking on bone accretion in late adolescence. The Tromsø study, Fit Futures

**DOI:** 10.1007/s11657-021-01003-7

**Published:** 2021-09-27

**Authors:** Ole Andreas Nilsen, Nina Emaus, Tore Christoffersen, Anne Winther, Elin Evensen, Gyrd Thrane, Anne-Sofie Furberg, Guri Grimnes, Luai Awad Ahmed

**Affiliations:** 1 Department of Health and Care Sciences, UiT The Arctic University of Norway, Tromsø, Norway; 2 Finnmark Hospital Trust, Alta, Norway; 3 Division of Neurosciences, Orthopedics and Rehabilitation Services, University Hospital of North Norway, Tromsø, Norway; 4 Department of Clinical Research, University Hospital of North Norway, Tromsø, Norway; 5 Molde University College, Molde, Norway; 6 Department of Microbiology and Infection Control, University Hospital of North Norway, Tromsø, Norway; 7 Division of Internal Medicine, University Hospital of North Norway, The Arctic University of Norway, Tromsø, Norway; 8 Endocrine Research Group, Department of Clinical Medicine, The Arctic University of Norway, Tromsø, Norway; 9 Institute of Public Health, College of Medicine and Health Sciences, United Arab Emirates University, Al Ain, United Arab Emirates

**Keywords:** Bone mineral density, DXA, Snuff use, Smoking, Adolescence, Osteoporosis

## Abstract

***Summary*:**

Areal bone mineral density (aBMD) predicts future fracture risk. This study explores associations between use of tobacco and bone accretion in Norwegian adolescents. Our results indicate that use of snuff is negatively associated with accretion of aBMD in adolescence and may be a signal of increased future fracture risk.

**Purpose:**

Bone mineral accrual in childhood and adolescence is a long-term primary preventive strategy of osteoporosis. Areal bone mineral density (aBMD) is a surrogate measure of bone strength and a predictor of fracture risk. The aim of this population-based 2-year follow-up cohort study was to explore associations between use of snuff and smoking and changes (∆) in aBMD in Norwegian girls and boys aged 15–17 years at baseline.

**Methods:**

The first wave of the Tromsø study, Fit Futures was conducted from 2010 to 2011. Femoral neck (FN), total hip (TH), and total body (TB) bone mineral content (BMC) and aBMD were measured by dual-energy X-ray absorptiometry. Information on use of snuff, smoking habits, and other lifestyle related variables were collected through self-administered questionnaires. Two years later, during 2012–2013, the measurements were repeated in the second wave. The present study included 349 girls and 281 boys and compared “non-users” (*n* = 243 girls, 184 boys) with “users” (*n* = 105 girls, 96 boys) of snuff and “non-smokers” (*n* = 327 girls, 249 boys) with “smokers” (*n* = 21 girls, 31 boys) using linear regression adjusted for age, baseline height and weight, change in height and weight, pubertal maturation, physical activity, ethnicity, alcohol consumption, diagnosis known to affect bone, and medication known to affect bone. The influence of “double use” on bone accretion was also explored.

**Results:**

In girls, no associations between use of snuff and ∆aBMD were found. In boys, use of snuff was associated with reduced bone accretion in all ∆aBMD models. Sensitivity analysis with exclusion of “sometimes” users of snuff strengthened associations at femoral sites in girls and attenuated all associations in boys. In girls, no associations between smoking and ∆aBMD were found. In boys, only the association with TB ∆aBMD was significant in the fully adjusted models. In girls, “double users” analyses showed similar association to smoking. In boys, nearly all models showed statistically significant associations with a difference of ~ 1–2% in ∆aBMD between “non-users” and “double users” during 2 years of follow-up.

**Conclusions:**

Our results indicate that tobacco use in late adolescence could be detrimental to bone accretion and may be a signal of increased fracture risk in adult life.

**Supplementary Information:**

The online version contains supplementary material available at 10.1007/s11657-021-01003-7.

## Introduction

Osteoporosis and its clinical manifestation, fragility fractures, constitute major public health challenges worldwide, and Norway has one of the highest reported hip fracture incidences in the world [1, 2]. Areal bone mineral density (aBMD) is a non-invasive way to assess bone strength and fracture risk [3]. Bone mineral levels in the elderly is a result of peak bone mass (PBM) achieved during childhood and adolescence and subsequent age-related bone loss [4]. Adolescence is a critical period for building bone as bone accrual peaks around the age of 12.5 among girls and 14 years of age for boys and roughly 95% of total adult bone mass is accrued within 4 years following the peak [5, 6]. Optimizing the genetic potential of PBM in adolescence is a long-term primary preventive strategy of osteoporosis. Around 20–40% of PBM achievement is attributed to lifestyle choices, and several modifiable behavioral determinants such as physical activity, body composition, and use of recreational drugs have been identified [7]. Tobacco use has been associated with lower aBMD during bone-building years [8, 9].

Over the past decades, there has been an increase in the prevalence of snuff users in Norway [10]. Snuff (Swedish snus) is smokeless, oral tobacco traditionally produced and mainly consumed in the Nordic countries. Within the EU/EEA area, Norway and Sweden are currently the only two countries allowing snuff for sale. However, even though the sale of snuff is prohibited in Finland and Denmark, data collected from these countries shows that snuff is also used there [11].

The increase in use of snuff in Norway started around the year 1990 among young men and after 2005 among women as well [12]. Whereas the amount of traditional smoking has decreased, snuff is now the most commonly used tobacco in the age range 16–24 years of age. The prevalence of daily smokers in these age groups has dropped from around 30% to roughly 5% in the last 20 years, while recent studies estimate that 25% of men and 15% of women use snuff daily [13, 14]. The prevalence of daily use of snuff is still increasing, although the rapid growth in proportion has slowed during the recent years [10].

There are constituents of snuff with a potentially wide range of adverse health effects, but these issues are relatively unexplored and evidence is controversial [15]. However, snuff and smoke tobacco expose individuals to many of the same substances, and the adverse influence of smoking on bone health in the adult population is well established [16]. The evidence of similar effects of tobacco on achievement of PBM during growth is suggestive, however, not compelling [7–9, 17–20]. Winther and colleagues found a negative association between smoking and aBMD in boys but no relationship between use of snuff and bone mass among Norwegian adolescents 15 to 17 years of age [21]. Apart from this cross-sectional study, the associations between snuff and bone at a population level are hardly described.

We hypothesized that both use of snuff and smoking may influence accretion of bone in adolescence. The aim of this study was therefore to explore possible association between use of snuff and change in aBMD (ΔaBMD) during a 2-year follow-up in late adolescence. In addition, the associations between smoking and “double use” and changes in bone traits were explored.

## Methods

### Subjects

The study procedures of The Tromsø study, Fit Futures are published previously [22]. Briefly, the Fit Futures study is a school-based cohort initiated in 2010–2011 (TFF1). All first year upper-secondary school students in Tromsø and the neighbor municipalities were invited to a comprehensive health survey. Out of 1117 invited individuals, 1038 adolescents (508 girls and 530 boys) attended, giving a participation rate of 92.9%. In the follow-up survey, Fit Futures 2 (TFF2) 2 years later (2012–2013), all participants in TFF1 and all new students in third year of the same upper-secondary schools were invited. A total of 66% of the TFF1 cohort met in TFF2 providing 688 repeated measures of aBMD. Participants above 17 years of age at baseline were excluded and 630 individuals 15 to 17 years of age, 349 girls and 281 boys, completed the questions on use of tobacco at both surveys.

Recruitment of participants to both surveys was conducted in close collaboration with the schools. The Clinical Research Unit at the University Hospital of North Norway conducted both health examinations during school days. All participants gave written informed consent at the study site. Participants younger than 16 years of age had to bring a written consent from their superiors to attend the survey. The data collection in TFF1 and TFF2 was approved by the Norwegian Data Protection Authority and the Regional Committee of Medical Research Ethics (REK nord) with a project-specific approval for the present study (Ref. 2019/31193/REK nord).

### Outcome measurements

Changes in bone mineral status were measured by dual-energy X-ray absorptiometry (DXA) as femoral neck (FN), total hip (TH), and total body (TB) bone mineral content (BMC; g) and aBMD (g/cm^2^). The same instrument (GE Lunar prodigy, Lunar Corporation, Madison, WI, USA) and analytic program (Encore pediatric software [23]) were used in both TFF1 and TFF2. We used auto-analysis software and default region of interest, according to a standardized protocol. Previously, the coefficients of variation ((SD/mean] × 100) for the DXA device used have been estimated to 1.14% at the TH and 1.72% at the FN measured in vivo [24]. We used measurements of the left-sided hip, but in 15 cases, the data was erroneous or missing, and values of the right hip were reported for both TFF1 and TFF2. The main outcome of this study was ΔaBMD; however, ΔBMC is frequently reported to support the understanding of bone accretion.

### Exposure variables

We collected data on the use of tobacco by electronic self-administered questionnaires. At TFF1, the question “Do you use snuff?” had three alternatives: “no never,” “sometimes,” or “daily.” If answers were “sometimes” or “daily,” participants were asked additional questions on frequency. The questions were as follows: “If you use snuff sometimes, how many snuff portions do you usually take per week?” Alternatives were “one or less,” “2–3,” “4–6,” “7–10,” and “more than 10.” For daily users, the subsequent question was as follows: “If you use snuff daily, how many snuff portions do you usually take per day?” Alternatives were “1,” “2–3,” “4–6,” “7–10,” and “more than 10.” Information about the age of onset were elicited by a question at TFF2: How old were you when you started to use snuff? The 8 alternatives were as follows: “Below 12 years,” “12 years,” “13 years,” “14 years,” “15 years,” “16 years,””17 years,” “18 years,” and “19 years or above.” Questions on smoking had an identical structure as those on use of snuff at both surveys, and only “portions” were replaced by “cigarettes.”

### Covariates

We measured height and body weight to the nearest 0.1 cm and 0.1 kg (Jenix DS 102 Stadiometer, Dong Sahn Jenix, Korea), following standardized procedures with no shoes and light clothing. Based on these parameters, body mass index (BMI) was calculated (kg/m^2^). Through clinical interviews, we assessed ethnicity (White, Asian, or Black, dichotomized into white or others), the possibility of pregnancy (exclusion criterion for DXA), acute and chronic diseases, use of medication, and use of hormonal contraceptives.

Pubertal maturation information, physical activity level, and alcohol consumption were elicited by the self-administered questionnaire at TFF1. Frequency of alcohol consumption was assessed with a scale from 1 to 5: “never,” “once per month or less,” “2–4 times per month,” “2–3 times per week,” and “4 or more times per week.” We dichotomized the answers into no (never) and yes. Covariates of pubertal maturation in boys were based on the Pubertal Developmental Scale (PDS). Secondary pubertal characteristics as growth spurt, pubic hair growth, changes in voice, and facial hair growth rated on a scale from 1 (have not begun) to 4 (completed) were summarized and divided by four [25]. In girls, pubertal maturation was determined based on self-reported age at menarche.

Self-reported physical activity level was assessed by questions from the modernized Saltin-Grimby Physical Activity Level Scale (SGPALS) [26]. The participants graded their leisure time physical activity in an average week during the last year with four alternatives: “sedentary activities only”; “moderate activity like walking, cycling, or exercise at least 4 h per week”; “participation in recreational sports at least 4 h per week”; “participation in hard training/sports competitions several times a week.” If the activity varied much, for example, between summer and winter, they were asked to give an average.

Hormonal contraceptive use (girls) was categorized into “no,” “estrogen and progestin,” and “progestin only.” We dichotomized answers on use of medication known to affect bone and diseases known to affect bone into yes and no (medication and disease definition, see Table 1).

**Table 1:** Characteristics at baseline survey Fit Futures 1 (TFF1) and annual change in height, weight, areal bone mineral density, and bone mineral content between TFF1 and Fit Futures 2 (TFF2) by use of snuff status: continuous variables presented as mean (standard deviation) and categorical variables in count (percentage). The Tromsø study, Fit Futures

		Girls							Boys						
		“Non-users” *n* = 244		“Sometimes” *n* = 51		“Daily users” *n* = 54		p	“Non-users” *n* = 185		“Sometimes” *n* = 31		“Daily users” *n* = 65		*p*
Age at baseline		16.61	(.36)	16.58	(.38)	16.67	(.48)	.483	16.61	(.35)	16.51	(.46)	16.60	(.37)	.404
Body height (cm)		165.44	(6.64)	164.21	(6.24)	164.16	(5.92)	.252	177.48	(6.59)	176.34	(6.24)	177.53	(6.66)	.651
Body weight (kg)		60.43	(10.96)	61.08	(9.39)	59.88	(10.06)	.844	68.94	(12.78)	71.11	(17.34)	71.23	(14.75)	.434
BMI, (kg/m2)		22.08	(3.80)	22.72	(3.93)	22.20	(3.45)	.546	21.85	(3.59)	22.80	(4.98)	22.56	(4.33)	.274
Maturation ^a^ Menarche age/PDS score		13.02	(1.17)	12.88	(1.14)	13.02	(1.28)	.734	3.26	(.43)	3.32	(.45)	3.42	(.43)	.65
Ethnicity white/others^b^		236	(96.7%)	51	(100.0%)	54	(100.0%)	.311	183	(98.9%)	30	(96.8%)	65	(100.0%)	.414
Physical activity	Sedentary	24	(9.8%)	2	(3.9%)	15	(27.8%)		42	(22.7%)	8	(25.8%)	23	(35.4%)	
	Moderate	95	(38.9%)	18	(35.3%)	26	(48.1%)		51	(27.6%)	9	(29.0%)	12	(18.5%)	
	Sports	87	(35.7%)	16	(31.4%)	6	(11.1%)		47	(25.4%)	8	(25.8%)	14	(21.5%)	
	Competition	38	(15.6%)	15	(29.4%)	7	(13.0%)	< .001	45	(24.3%)	6	(19.4%)	16	(24.6%)	.505
Portions of snuff weekly/daily^c^	< = 1			28	(54.9%)	0	(0.0%)				18	(58.1%)	1	(1.5%)	
	2–6			16	(31.4%)	27	(50.0%)				5	(16.1%)	28	(43.1%)	
	> = 7			7	(13.7%)	27	(50.0%)				8	(13.7%)	36	(55.4%)	
Do you smoke?	No never	226	(92.6%)	33	(64.7%)	26	(48.1%)		177	(95.7%)	19	(61.3%)	33	(50.8%)	
	Sometimes	17	(7.0%)	15	(29.4%)	24	(44.4%)		8	(4.3%)	9	(29.0%)	27	(41.5%)	
	Daily	1	(0.4%)	3	(5.9%)	4	(7.4%)	< .001	0	(0.0%)	3	(9.7%)	5	(7.7%)	< .001
Cigarettes weekly/daily^c^	< = 1			43	(76.8%)	0	(0.0%)				21	(47.7%)	0	(0.0%)	
	2–6			11	(19.6%)	6	(75.0%)				19	(43.2%)	4	(50.0%)	
	> = 7			2	(3.6%)	2	(25.0%)				4	(9.1%)	4	(50.0%)	
Do you drink alcohol? (yes)		151	(61.9%)	51	(100.0%)	54	(100.0%)	< .001	92	(49.7%)	29	(93.5%)	61	(95.3%)	< .001
Diagnosis (yes)^d^		4	(1.6%)	0	(0.0%)	0	(0.0%)	1.000	4	(2.2%)	0	(0.0%)	1	(1.5%)	1.000
Medication (yes)^e^		4	(1.6%)	3	(5.9%)	1	(1.9%)	.133	6	(3.2%)	0	(0.0%)	0	(0.0%)	.345
Hormonal contraceptives	No	186	(76.2%)	28	(54.9%)	19	(35.2%)								
	Estrogen and progestin	52	(21.3%)	22	(43.1%)	29	(53.7%)								
	Progestin only	6	(2.5%)	1	(2.0%)	6	(11.1%)	< .001							
aBMD FN (g/cm^2^)		1.071	(.117)	1.091	(.155)	1.051	(.122)	.252	1.108	(.150)	1.128	(.170)	1.113	(.148)	.798
aBMD TH (g/cm^2^)		1.061	(.114)	1.090	(.156)	1.038	(.136)	.101	1.121	(.147)	1.143	(.171)	1.119	(.151)	.738
aBMD TB (g/cm^2^)		1.142	(.074)	1.148	(.084)	1.123	(.069)	.167	1.176	(.092)	1.192	(.100)	1.194	(.101)	.349
BMC FN (g)		4.936	(.691)	4.976	(.852)	4.785	(.587)	.296	5.966	(.962)	6.121	(1.126)	6.040	(1.031)	.676
BMC TH (g)		32.137	(4.713)	32.821	(5.393)	31.025	(4.738)	.149	39.980	(6.457)	41.382	(7.546)	40.460	(6.747)	.531
BMC TB (g)		2532.361	(387.830)	2548.507	(370.368)	2481.440	(394.242)	.620	2940.101	(445.767)	2989.046	(530.478)	3021.854	(517.739)	.466
ΔBody height (cm per year)		.38	(.47)	.24	(.41)	.42	(.36)	.073	1.06	(.89)	.91	(.88)	.62	(.74)	.002
ΔBody weight (kg per year)		1.47	(2.59)	1.20	(2.43)	1.15	(2.29)	.590	3.00	(3.05)	1.99	(2.91)	2.01	(2.88)	.031
ΔaBMD FN (g/cm^2^ per year)		.004	(.019)	.003	(.018)	− .004	(.019)	.019	.022	(.026)	.003	(.024)	.010	(.027)	< .001
ΔaBMD TH. (g/cm^2^ per year)		.007	(.017)	.005	(.016)	− .001	(.015)	.012	.016	(.022)	.002	(.022)	.005	(.021)	< .001
ΔaBMD TB (g/cm^2^ per year)		.009	(.010)	.008	(.010)	.008	(.009)	.679	.026	(.015)	.019	(.013)	.017	(.014)	< .001
ΔBMC FN (g per year)		.022	(.097)	.012	(.087)	− .013	(.091)	.047	.132	(.174)	.010	(.194)	.055	(.156)	< .001
ΔBMC TH (g per year)		.227	(.591)	.107	(.621)	.038	(.537)	.066	.772	(1.080)	.067	(.997)	.260	(.984)	< .001
ΔBMC TB (g per year)		39.680	(60.349)	47.630	(66.963)	27.898	(55.011)	.238	132.075	(81.248)	97.151	(60.081)	94.687	(69.825)	.001
Time between measurements (years)		1.925	(.198)	1.935	(.159)	2.055	(.211)	< .001	1.949	(.214)	2.078	(.218)	2.103	(.213)	< .001

Δ = change. *aBMD* areal bone mineral density, *BMC* bone mineral content, *FN* femoral neck, *TH* total hip, *TB* total body. ^a^Missing PDS score *n* = 52. ^b^Percentage of white. ^c^Sometimes category shows units per week, and daily category shows units per day. ^d^Diseases known to affect bone (ICD10): E03 Hypothyroidism, E10 Diabetes type 1, F50,9 Eating disorders, K90.0 Celiac disease, and M13 Arthritis. ^e^Medication known to affect bone (ATC): D07A Plain corticosteroids, H03A Thyroid preparations, N03A Antiepileptic, R01AD Corticosteroids, R03BA Glucocorticoids (inhalants), and H02A Corticosteroids for systemic use. *p* = ANOVA

### Statistical analyses

All statistical analyses were conducted stratified by sex, and characteristics of the study population are presented as means and standard deviations (SD), or count, and percentages. We explored differences by ANOVA with Bonferroni correction and Pearson’s chi-squared test. We calculated absolute change (TFF2 − TFF1) and percentage change ((TFF2 − TFF1)/TFF1*100) in bone traits. Through DXA measurement dates, we were able to compute exact time of follow-up to compute annual change of anthropometric and bone parameters used in crude analyses. For simplicity purposes, the snuff and cigarette frequency answers were categorized into three groups: “ < 1,” “2–6,” and “ > 7” units per week/day.

The associations between the exposure of tobacco and the outcomes of ΔaBMD between TFF1 and TFF2 where investigated by linear regression models. We used TFF2 score as outcome and included the TFF1 score as a covariate to estimate the predictive value of exposure on change (*Y2* = *β0* + *β1Y1* + *β2X*_*snuff*_ + β3…). We compared the results with change-score analysis (*Y2*-*Y1* = *β 0* + *β1X*_*snuff*_) and explored consistency as baseline adjustments in change-score analysis may introduce bias in regression models comparing naturally occurring groups [27].

Initially we conducted crude models. Then potential confounders were added in the following way: model 2, the “anthropometry model,” comprised the crude model plus age, baseline anthropometry (height and weight), and change in anthropometric parameters. In model 3, the full model, pubertal maturation, and baseline physical activity level were added to the “anthropometry model.” In addition, baseline variables previously known to be of clinical importance like ethnicity, alcohol consumption, diagnosis known to affect bone, medication known to affect bone (see Table 1), and hormonal contraceptives use (girls) were then added as covariates using a backwards elimination strategy where *p* = 0.10 were used as cut-off to stay or leave the model. Based on these elimination procedures, ethnicity was added to all models in boys, and the TH ΔaBMD was adjusted for medication known to affect bone. In girls, hormonal contraceptives use was added to all models. All models were adjusted for time between measurements. Use of snuff models were controlled for daily smoking and vice versa.

During the first few weeks of TFF1, the questionnaire did not contain the questions related to PDS score, giving a high percentage of missing puberty values among boys: *n* = 52 (18.5%). In girls, six had missing information on menarche age. Multiple imputations based on predictors and outcome variables used in the full model were conducted to predict missing values. We assumed missing at random, 20 imputations were performed, and we report pooled estimates [28]. Normal distribution, linearity, homogeneity, and outliers were explored by residual analysis. In both girls and boys, one outlier was excluded in all models. In girls, the full TB BMC model residuals showed a heteroscedastic pattern, and weighted least square regression was applied. We used menarche age and PDS scores as continuous variables in multiple regression models. Plausible 2-way interactions related to aBMD, age, and pubertal maturation were checked, and interaction terms for age*snuff were added to boys ΔaBMD TB full model and age* “double use” to the ΔaBMD TB full model in girls. Significance level was set to *p* = 0.05, and all procedures were performed in IBM SPSS Statistics for Windows, version 26 (IBM Corp., Armonk, N.Y., USA).

## Results

We included 630 adolescents in the present study, 349 girls and 281 boys (45% boys), and their descriptive statistics stratified by use of snuff status are presented in Table 1. Mean age of the participants at baseline was 16.6 (SD 0.4) years with a range from 15.7 to 17.9 years. The majority were 16 years of age, 83.1% and 80.8% in girls and boys, respectively. At follow-up, the mean age was 18.6 (SD 0.4) years with a range from 17.8 to 20.1 years. Mean follow-up time between TFF1 and TFF2 was 2.0 (SD 0.2) years with a range between 1.5 and 2.7 years. Only participants with repeated measures were included in the study and drop-out analysis revealed that significantly more boys (*n* = 196) than girls (*n* = 111) were lost to follow-up. Girls lost to follow-up had higher BMI (0.894 kg/m^2^, *p* = 0.039), while boys had lower baseline FN aBMD (− 0.028 g/cm^2^, *p* = 0.049). Both girls and boys lost to follow-up had a statistically significant higher prevalence of snuff use (girls, *p* = 0.001; boys, *p* = 0.005) and smoking (girls, *p* = 0.013; boys, *p* = 0.032).

### Use of snuff

In girls, 244 (69.9%) were classified as “non-users,” 51 (14.6%) as “sometimes,” and 54 (15.5%) as “daily users” of snuff at baseline. In boys, the corresponding numbers were 185 (65.9%), 31 (11.0%), and 65 (23.1%), respectively. The age of onset was mainly between 13 and 16 years of age, and mean age was 14.6 in girls (*n* = 93, 12 missing) and 14.7 years in boys (*n* = 84, 12 missing; 3 participants responding onset under 12 years had age set to 11 years). The age of onset was not correlated with any bone outcomes.

All but six of the “daily users” at TFF1 remained “daily users” during follow-up (51 girls and 62 boys at TFF2). The prevalence of snuff users increased during follow-up. In both girls and boys, roughly 18% “of the non-users” reported sometimes or daily use of snuff 2 years later. In the “sometimes” use of snuff category at baseline, more than half of the girls reported to usually take one portion or less per week and 86.3% reported six portions per week or less. In boys, we observed corresponding numbers, with 58.1% and 74.2%, respectively. Furthermore, we observed that individuals in the “sometimes” category at baseline fluctuated during follow-up, and only 15 of the 82 remained in their initial “sometimes” category. In main analysis, we compared the “non-users” with the “users” of snuff, combining “sometimes” and “daily” users of snuff at baseline. Sensitivity analysis was then conducted with the “sometimes” group excluded, comparing “non-users” with “daily users” group only.

In both girls and boys, the snuff “users” group differed significantly from the “non-users” with a higher prevalence of smokers and alcohol consumers (*p* < 0.001). Among girls in the “users” category, fewer reported to be engaged in sports activities (*p* < 0.001), and use of hormonal contraceptives was more prevalent in the “users” group (*p* < 0.001). In boys, there was a statistically significant difference between the compared groups in annual height (*p* < 0.001) and weight change (*p* = 0.020). No differences in baseline aBMD or BMC between the groups were observed.

### Crude analyses

**Fig. 1:**
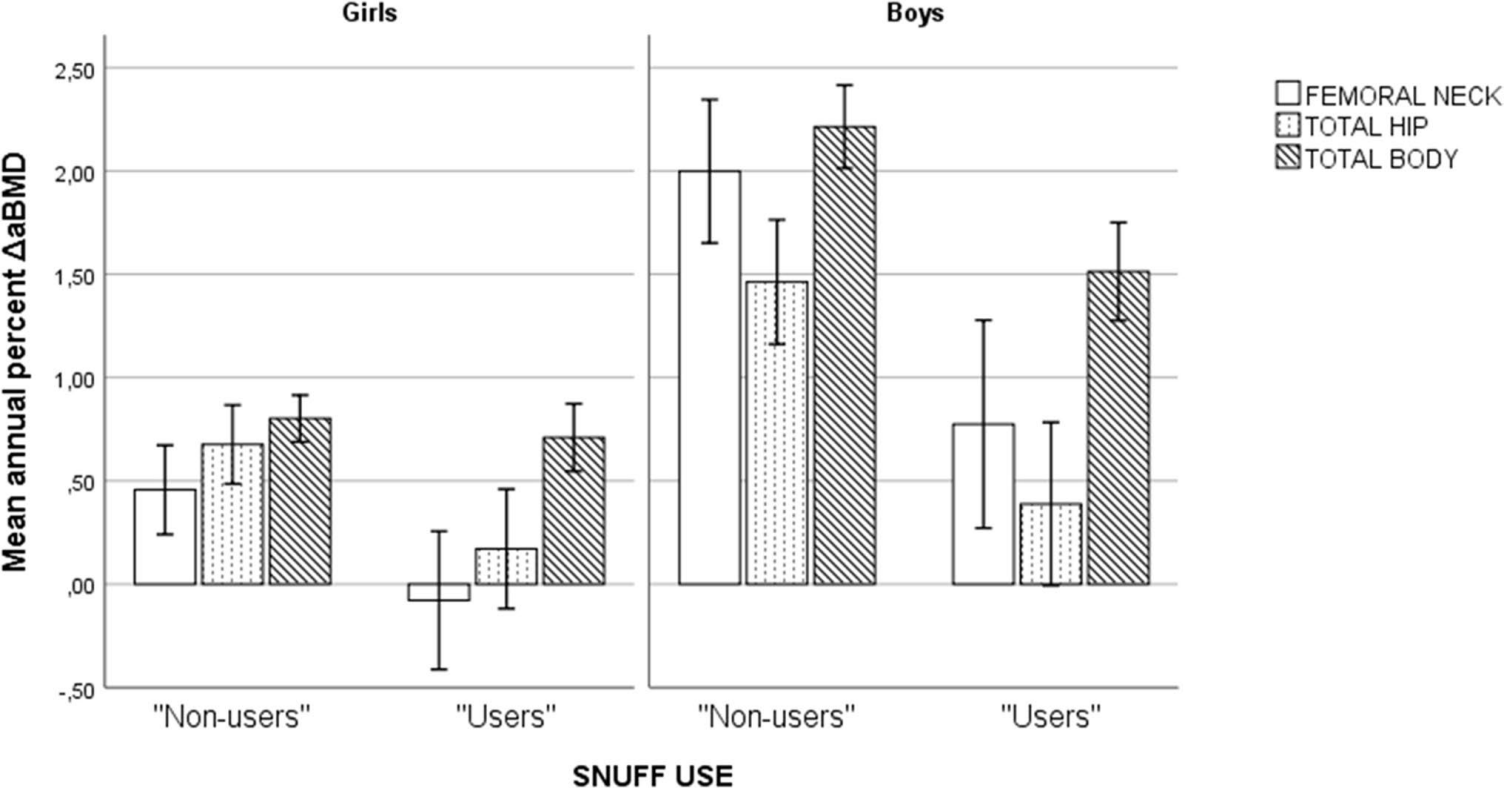
Crude comparisons of “non-users” and “users” of snuff with regard to mean annual percent change in Areal bone mineral density (aBMD) between baseline and follow-up measurement 2 years later for femoral neck, total hip, and total body. The Tromsø study, Fit Futures. Girls, *N* = 349 (244 “non-users”/105 “users”). Boys, *N* = 281 (185 “non-users”/96 “users”)

Crude percentage of bone accretion is shown in Fig. 1. In both girls and boys reporting no use of snuff at baseline, mean annual ΔaBMD was higher compared to change among “users.”

### Adjusted analyses

The results of crude and adjusted regression models of ΔaBMD and ΔBMC in relation to use of snuff are presented in Table 2. In girls, the “users” of snuff group had significantly less ΔaBMD compared to the “non-user” group in crude models at both femoral sites (FN: β =  − 0.004, *p* = 0.028, and TH: β =  − 0.019, *p* = 0.020), but not at TB. No associations were significant in the fully adjusted models.

**Table 2:** Crude and adjusted associations between snuff use at baseline (use versus non-use) and change in femoral neck (FN), total hip (TH), and total body (TB) areal bone mineral density and bone mineral content during about 2 years follow-up, adjusted for baseline measurement. The Tromsø study, Fit Futures

	Use of snuff	FN		TH		TB	
		*β*	*p*	*β*	*p*	*β*	*p*
Girls							
ΔaBMD	Crude	** − .004**	**.028**	** − .009**	**.020**	** − **.002	.418
	Age and anthropometry	** − **007	.084	** − **.007	.063	** − **.001	.793
	Full model	** − **.004	.304	** − **.003	.443	.000	.862
ΔBMC	Crude	** − .045**	**.036**	** − .310**	**.018**	** − **1.848	.893
	Age and anthropometry	** − **.034	.100	** − **.245	.052	10.107	.404
	Full model	** − **.073	.057	** − **.122	.357	** − **9.454 §	.783
Boys							
ΔaBMD	Crude	** − .024**	**.000**	** − .019**	**.001**	** − .015**	**.000**
	Age and anthropometry	** − .017**	**.009**	** − .013**	**.014**	** − .009**	**.010**
	Full model	** − .015**	**.023**	** − .012 #**	**.027**	** − .322 ¤**	**.019**
ΔBMC	Crude	** − .170**	**.000**	** − 1.095**	**.000**	** − 74.395**	**.000**
	Age and anthropometry	** − .120**	**.004**	** − .712**	**.003**	** − **25.073	.084
	Full model	** − .099¤**	**.020**	** − .779**	**.007**	** − **25.021	.098

Girls, *N* = 348 (243 “non-users”/105 “users”). Boys, *N* = 280 (184 “non-users”/96 “users”). Values are based on linear regression analysis. Δ = change. *aBMD* areal bone mineral density, *BMC* bone mineral content. Anthropometry model: crude model + age, body height, body weight, Δbody height, and Δbody weight. Full model: anthropometry model + pubertal maturation, physical activity level, daily smoking, hormonal contraceptives use (girls only), and ethnicity (boys only). All models were adjusted for time between measurements. ^#^Adjusted for medication known to affect bone. ^§^*n* = 342 because of weighted least square model. ^¤^Interaction age*snuff use β = .019, *p* = .022. Statistically significant results in bold

In boys, statistically significant associations were observed in both ΔaBMD and ΔBMC, except in the adjusted ΔBMC TB models. Estimated ΔaBMD between “non-users” and “users” of snuff at the FN was 0.012 and 0.015 g/cm^2^ at the TH in the full models, a difference comprising roughly 1% change during follow-up. In anthropometry models, particularly changes in anthropometric measures attenuated the associations.

### Sensitivity analyses

In analysis where the “sometimes” group was excluded, comparing “non-users” (girls, *n* = 244; boys, *n* = 185) with “daily users” (girls, *n* = 54; boys, *n* = 65) of snuff showed estimates with negative associations between snuff use and bone accrual. In girls, the “daily users” of snuff group had significantly less ΔaBMD at FN compared to the “non-user” group in adjusted models: β =  − 0.012, *p* = 0.037, while the TH association also strengthened (β =  − 0.009, *p* = 0.071). In boys, both ΔaBMD and ΔBMC crude models were statistically significant. However, in partly adjusted models, all associations were attenuated and turned out insignificant. In the fully adjusted models, use of snuff was not statistically significantly associated with ΔaBMD.

In sensitivity analysis related to multiple imputation and the high percentage of missing puberty data in boys, the full models with the original sample (“non-users” vs “users” with original PDS score, *n* = 229) showed similar estimates, but FN (β =  − 0.015, *p* = 0.059) and TB (β =  − 0.008, *p* = 0.062) turned out insignificant.

### Smoking and bone accretion

There were a limited number of daily smokers at baseline in the study population, eight girls (2.3%) and eight boys (2.8%). In the “sometimes” category consisting of 56 girls (16%) and 44 (15.7%) boys, 76.8% of the girls and 47.7% of the boys reported to smoke one or less cigarette a week. In order to obtain a larger comparison group and to enhance exposure of smoking in the “smokers” category, the “one or less” responders were regarded as “non-smokers,” and a combined “daily/more than 2 cigarettes weekly” category consisting of 21 girls and 31 boys was created.

**Fig. 2:**
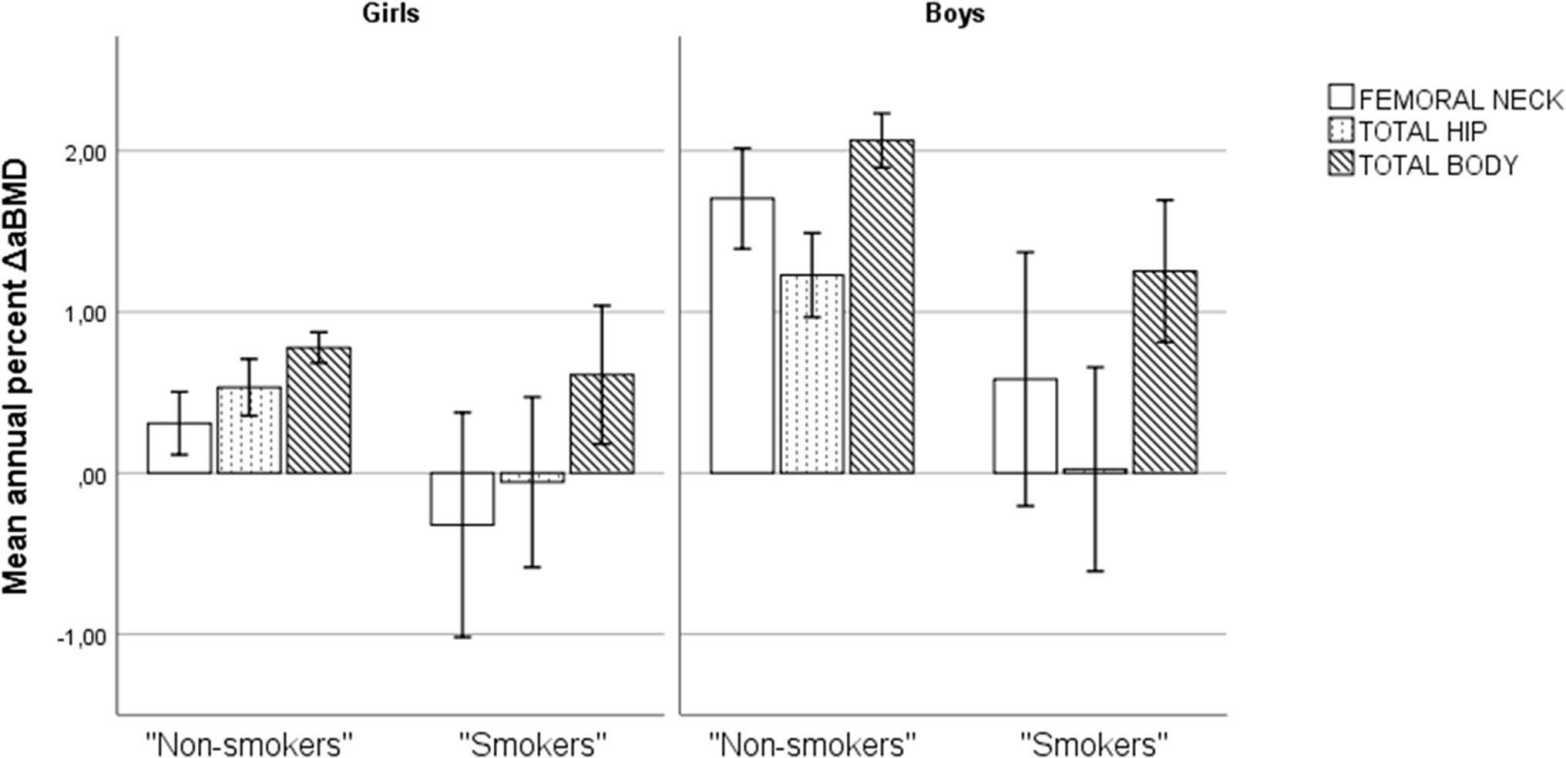
Crude comparisons of “non-smokers” and “smokers” with regard to mean annual percent ΔaBMD between baseline and follow-up measurement 2 years later for femoral neck, total hip, and total body. The Tromsø study, Fit Futures. Girls, *N* = 349 (228 “non-users”/21 “users”). Boys, *N* = 281 (250 “non-users”/31 “users”)

No statistically significant differences in baseline bone traits between smokers and “non-smokers” were observed. Otherwise, the groups differed with a higher prevalence of snuff use, alcohol consumption, and lower physical activity levels in the smokers group compared to the “non-smokers” group. Crude comparisons of change in ΔaBMD are shown in Fig. 2.

The results of crude and adjusted regression models of ΔaBMD and ΔBMC in relation to smoking are presented in Table 3. In girls, no associations between smoking and ΔaBMD were observed, but accumulation of BMC at the FN appeared to be reduced (β = 0.109, *p* = 0.006).

**Table 3:** Crude and adjusted associations between smoking status (smoker versus non-smoker) at baseline and follow-up measurement 2 years later for femoral neck (FN), total hip (TH), and total body (TB) aBMD and BMC, adjusted for baseline measurement. The Tromsø study, Fit Futures

	Smoking	FN		TH		TB	
		*β*	*p*	*β*	*p*	*β*	*p*
Girls							
ΔaBMD	Crude	− .011	.121	− .011	.128	− .004	.322
	Age and anthropometry	− .011	.142	− .013	.063	− .004	.344
	Full model	− .009	.288	− .009	.206	− .003	.521
ΔBMC	Crude	− **.096**	**.019**	− .343	.176	.342	.990
	Age and anthropometry	− **.105**	**.008**	− **.487**	**.046**	− 11.898	.613
	Full model	− **.097**	**.012**	− .348	.169	− 22.099*	.264
Boys							
ΔaBMD	Crude	− .009	.398	− **.021**	**.016**	− **.016**	**.006**
	Age and anthropometry	− .011	.225	− **.013**	**.006**	− **.013**	**.008**
	Full model	− .009	.411	− .016	.052	− **.011**	**.037**
ΔBMC	Crude	− **.165**	**.014**	− **1.127**	**.004**	− 49.356	.108
	Age and anthropometry	− **.125**	**.042**	− **.945**	**.007**	− 32.578	.123
	Full model	− .094	.145	− .752	.050	− 24.040	.293

Girls, *N* = 348 (327 “non-smokers”/21 “smokers”). Boys, *N* = 280 (249 “non-smokers”/31 “smokers”).Values are based on linear regression analysis. Δ = change. *aBMD* areal bone mineral density, *BMC* bone mineral content. Anthropometry model: crude model + age, body height, body weight, Δbody height, and Δbody weight. Full model: age and anthropometry model + pubertal maturation, physical activity level, daily snuff use, and ethnicity (boys only). All models were adjusted for time between measurements.*n = 342 because of weighted least square model. Statistically significant results in bold

In boys, the full ΔaBMD TB model was statistically significant (β =  − 0.011, *p* = 0.037). The association for TH in the crude and anthropometric models was statistically significant but was attenuated in the full model with a borderline association (*p* = 0.052).

### “Double use”

At baseline, 46 girls and 44 boys responded that they were “double users,” indicating responses of either “daily” or “sometimes,” for both smoking and use of snuff at baseline. Among the “daily users” of snuff, 28 (51.9%) of the girls and 32 (49.2%) of the boys reported to be “double users,” i.e., both smokers and daily use of snuff. These individuals were mostly sometimes smokers as only eight girls and eight boys reported smoking daily. Only one out of the 16 daily smokers at baseline was not “double user.”

The baseline differences between “double users” and “non-users” were similar to use of snuff, except that annual change in body weight did not differ in boys. The results of crude and adjusted regression models of ΔaBMD, ΔBMC, and “double use” are presented in Table 4.

**Table 4:** Crude and adjusted associations between double use of tobacco and change in femoral neck (FN), total hip (TH), and total body (TB) aBMD and BMC during 2 years follow-up, adjusted for baseline measurement. The Tromsø study, Fit Futures

	Double users	FN		TH		TB	
		*β*	*p*	*β*	*p*	*β*	*p*
Girls							
ΔaBMD	Crude	− .009	.123	− .006	.216	− .003	.293
	Age and anthropometry	− .010	.074	− .008	.114	− .003	.263
	Full model	− .009	.114	− .004	.370	− **.249 §**	**.020**
ΔBMC	Crude	− **.066**	**.024**	− .276	.122	2.794	.881
	Age and anthropometry	− **.073**	**.009**	− **.337**	**.048**	− 2.055	.900
	Full model	− **.068**	**.018**	− .210	.224	− 4.514 *	.728
Boys							
ΔaBMD	Crude	− **.021**	**.017**	− **.024**	**.001**	− **.018**	** < .001**
	Age and anthropometry	− .012	.128	− **.018**	**.005**	− **.013**	**.002**
	Full model	− .012	.124	− **.018**	**.007**	− **.013**	**.002**
ΔBMC	Crude	− **.161**	**.005**	− **1.127**	**.004**	− **64.944**	**.013**
	Age and anthropometry	− **.105**	**.045**	− **.933**	**.003**	− 32.578	.123
	Full model	− **.103**	**.047**	− **.898**	**.003**	− 32.191	.073

Girls, *N* = 348 (302 “non-users”/46 “double users”). Boys, *N* = 280 (236 “non-users”/44 “double users”).Values are based on linear regression analysis. Double users = snuffing and/or smoking daily. Δ = change. *aBMD* areal bone mineral density, *BMC* bone mineral content. Anthropometry model: crude model + age, body height, body weight, Δbody height, and Δbody weight. Full model: anthropometry model + pubertal maturation, baseline physical activity level, and ethnicity (boys only). All models were adjusted for time between measurements.*n = 342 because of weighted least square model.^§^Interaction age*double use β = .015, *p* = .021. Statistically significant results in bold

In girls, no relationship was observed between ΔaBMD and “double use,” while the ΔBMC was significantly reduced at the FN (*p* = 0.018). In boys, most models turned out statistically significant, except the adjusted ΔaBMD FN models. An estimated difference in ΔaBMD between “non-users” and “double users” at the TH of 0.018 g/cm^2^ corresponds to ~ 1.6% change during follow-up.

## Discussion

To our knowledge, this is the first population-based study to explore associations between use of snuff and bone accumulation in a young population. In girls, snuff use was not associated with bone accretion in main analyses, however with an indicated inverse association at femoral sites when “non-users” and “daily users” of snuff were compared. In boys, negative associations between use of snuff and ΔaBMD were observed at all skeletal sites. However, in contrast to girls, associations attenuated when comparing “non-user” and “daily users” of snuff in sensitivity analyses. Smoking had limited influence on ΔaBMD, while “double use” was associated with a lower rate of bone accumulation at TH and TB during follow-up among boys. With a few exceptions, the regression coefficients were negative for both use of snuff, smoking, and “double use.” However, the statistical significance of the associations was not consistent and depended on skeletal site and sex.

The use of snuff in a young population is a relatively new public health issue in Scandinavia, and the health-related effects of snuff are not much studied. Winther and colleagues explored the cross-sectional associations between aBMD and use of snuff in the TFF1 study population and found no statistically significant differences between “users” and “non-users” [21]. The absence of a relationship may be explained by age of onset, duration of use, and temporal sequence of events. The majority of daily users of snuff reported onset to be 1 or 2 years before participation in TFF1, and the influence of snuff on bone mass may not have been established yet.

In a study from 2007, no delayed bone healing was observed in male users of snuff after osteotomy [29]. Some studies have shown that snuff use status and periodontal bone loss are related [30], but these findings concerning the oral cavity in adults are not necessarily comparable to skeletal health, nor valid in an adolescent population. There are some studies showing that smokeless tobacco (chewing tobacco, non-combustible tobacco) accelerates age-related loss of aBMD in various populations, typically in India [31], Turkey [32], and in older multi-ethnic women [33, 34]. However, it has been argued that Swedish snuff has a lower potential of harm due to reduced levels of chemical agents than tobacco products consumed by populations in other geographical areas worldwide [35] and, thus, may not be comparable with these other types of substances related to bone.

The differences in significance between girls and boys may be explained by differences in maturation and timing of PBM. The boys are still in longitudinal growth, and the hypothesized detrimental influence of use of tobacco may have a greater impact compared to girls. A previously published study of the Fit Futures cohort has shown that girls are reaching an aBMD plateau at femoral sites in late adolescence [22].

Individuals that use tobacco may be more likely to have a lifestyle that could negatively affect aBMD than their “non-user” peers, and it may be hard to disentangle these factors. Essential confounders like body weight and physical activity were adjusted for in the full models. Together with changes in anthropometry, smoke was the major confounder of the relationship between bone and use of snuff, and the same tendency was observed for snuff on smoke. This gave the grounds for analysis of double users.

One crucial confounder in this study is the influence of pubertal maturation. The developmental differences of normal puberty are large, and hormonal status influences timing of bone accretion. The rate of bone accretion largely depends on biological rather than chronological age [5]. In girls, there was a dose–response relationship between “no never,” “sometimes,” and “daily” use of snuff and ΔaBMD. In boys, the “sometimes” group gained less on average at femoral sites than the “daily users” did. This could explain why sensitivity analysis showed no differences between the “daily users” and “non-users” of snuff. The “sometimes group” did, however, have a higher initial aBMD value. The attenuation of the associations by changes in anthropometrics in boys could indicate that some of the variation in bone accretion is due to differences in maturation not explained by pubertal maturation variable PDS score. The precision of self-reported PDS score has been questioned [36], and use of snuff could be influenced by timing of pubertal maturation, as previously reported for smoking and alcohol consumption in another Norwegian cohort [37].

The influence of smoking on PBM has been investigated more thoroughly; however, the evidence is not compelling and limited by methodological challenges. Weaver et al. [7] identified 6 prospective and 7 cross-sectional studies published since year 2000 with inconsistent conclusions, but overall evidence supported the notion that smoking may have a deleterious influence on PBM. Discrepancy of associations may be due to diverse classifications of smoking status employed or frequency- and duration-dependent effects of smoking on bone. The low prevalence of regular smoking frequently limits statistical power [7]. Our study was no exception. Dorn and colleagues [8] found that the effect of smoking on bone accrual became more pronounced as girls got older. We could not confirm this relationship in the TFF cohort, but this may be related to sample size, low prevalence of smokers, and degree of exposure.

### Mechanisms

Potential pathophysiologic mechanisms of the adverse effects of tobacco on bone remain to be clarified [38]. Snuff may have different effects on bone than smoking does, because it does not undergo combustion. Nevertheless, the hypothesized influence of tobacco on the skeleton may be indirect through body weight, reduced blood supply, calciotropic hormones, and abnormal PTH/vitamin D axis or direct through nicotine [38–40]. Studies suggest that snuff generates the same amount of blood plasma nicotine level as smoking [41]. There is a faster uptake of nicotine by smoking, but the blood plasma nicotine level remains higher over a longer period of time by use of snuff [42]. However, the influence of nicotine on bone may be different in growing and mature skeletons [43].

### Study strengths and limitations

The main strengths of this study are the population-based design and a relatively large, well-described, study population where both sexes are represented. The Tromsø study, Fit Futures is one of few studies investigating adolescent’s health and lifestyle. Trained personnel at the research unit at the University Hospital of North Norway conducted the data collection in order to minimize measurement error. However, the study has some limitations to consider. Only two measurement points and the relatively short follow-up time made it challenging to differ true change in aBMD from measurement error and regression to the mean may be an issue. Even though the precision of DXA is good at a group level, the mean changes of less than 1% are debatable given the CV of the DXA scanner. DXA has limitations because of the two-dimensional measurement of BMD, leading to overestimation of larger bones. The assessment of tobacco exposure by self-administered questionnaire may induce social desirability bias and underreporting of exposure [44]. The lack of information on nutrition and vitamin D levels and the missing PDS scores for 18.5% of males are considered a limitation to the study. Loss to follow-up bias may influence the validity of this study, as the study sample comprises roughly 66% of the original cohort. However, a high attendance rate at baseline (93%) contributed to the information of the drop-out analysis. Proportions of smokers and users of snuff were higher in drop-outs, which could lead to an underestimation of the associations between tobacco and bone accrual in the study. The use of baseline adjustments in two-wave observational studies with naturally occurring groups is debated. Baseline adjustments combined with measurement error may lead to directional bias leaving hypothesis tests vulnerable to type 1 error [45], and comparison with simple change scores without baseline adjustments was conducted according to advices by van Breukelen [27]. When the two approaches do not agree, results should be interpreted with caution. The disagreement was limited to 1 out of 36 models (Supplemental table). Nevertheless, disagreement may also partly be explained by the fact that one approach estimates the total effect of exposure on bone accrual, while the other estimates the direct effect, adjusting for the initial bone trait level [46].

## Conclusions

Our findings suggest an inverse association between use of snuff and aBMD changes in late adolescence. In girls, no differences between “non-users” and “users” were identified, but snuff use was associated with lower femoral ΔaBMD when comparing “non-users” with “daily users” of snuff only. In boys, negative associations between use of snuff and ΔaBMD were observed at all skeletal sites. However, in contrast to girls, associations attenuated when comparing “non-user” and “daily users” of snuff in sensitivity analyses. The associations between smoking and change in bone traits were limited, while combined use of cigarettes and snuff, “double use,” appeared to have a detrimental influence on bone accrual in boys. The results should be interpreted with caution due to limitations of the two-wave design, potentially unobserved pubertal maturation interactions, low prevalence of smokers, and a short follow-up time. However, the study findings partly support our hypothesis that the use of snuff and smoking are detrimental to bone accretion and should be investigated further in cohorts with multiple waves as the consumption of snuff is rising among the adolescent population and future bone health consequences are unclear.

## Supplementary Information

Below is the link to the electronic supplementary material.

Supplementary file1 (DOCX 23 kb)

Supplementary file2 (DOCX 25 kb)

Supplementary file3 (DOCX 16 kb)
