# Supplementary material for: The influence of snuff and smoking on bone accretion in late adolescence. The Tromsø study, Fit Futures
Source: Arch Osteoporos. 2021 Sep 27;16(1):143. doi: 10.1007/s11657-021-01003-7 (PMC8476466; doi:10.1007/s11657-021-01003-7)
Supplement: Supplementary file 1 — Supplementary file1 (DOCX 23 kb) [file 11657_2021_1003_MOESM1_ESM.docx]

**Supplemental figure 1** Flowchart of participation in Fit Futures 1 (TFF1) 2010-2011 and Fit Futures 2 (TFF2) 2012-2013.

1301 Registered students for first year upper secondary school 2010/11.

1117 was invited to TFF1.

1038 participated TFF1.

508 girls and 530 boys.

Not reached by invitation/dropped out of school: n=184

Did not attend TFF1: n=79

Excluded:

>17 years at baseline n=34.

Missing data outcome variables n=3. Missing data on tobacco n= 21.

Lost to follow-up: n=350

688 repeated DXA measures in TFF2

(66% of the TFF1 cohort).

349 girls and 281 boys with complete dataset
