# Supplementary material for: The influence of snuff and smoking on bone accretion in late adolescence. The Tromsø study, Fit Futures
Source: Arch Osteoporos. 2021 Sep 27;16(1):143. doi: 10.1007/s11657-021-01003-7 (PMC8476466; doi:10.1007/s11657-021-01003-7)

***Figure 2.*** *Distribution of age when starting to use snuff (“users”). The Tromsø Study, Fit Futures. Girls N =93. Boys N =84.*


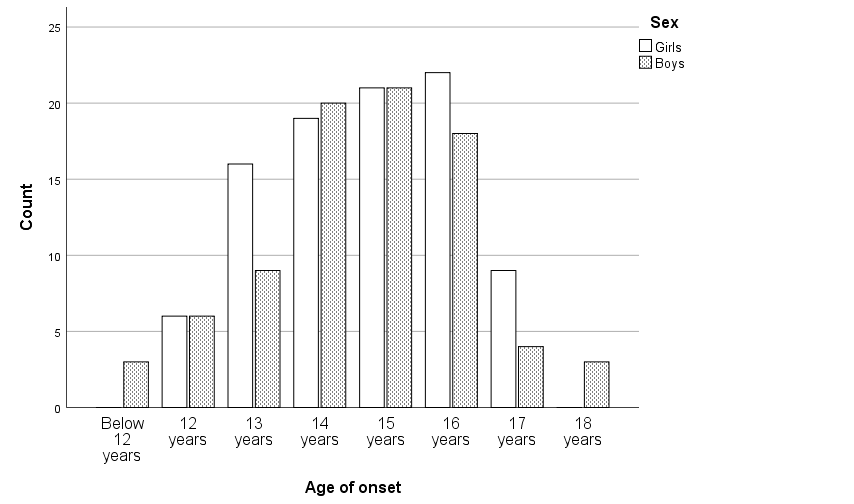

Supplement: Supplementary file 2 — Supplementary file2 (DOCX 25 kb) [file 11657_2021_1003_MOESM2_ESM.docx]
