# Supplementary material for: The influence of snuff and smoking on bone accretion in late adolescence. The Tromsø study, Fit Futures
Source: Arch Osteoporos. 2021 Sep 27;16(1):143. doi: 10.1007/s11657-021-01003-7 (PMC8476466; doi:10.1007/s11657-021-01003-7)
Supplement: Supplementary file 3 — Supplementary file3 (DOCX 16 kb) [file 11657_2021_1003_MOESM3_ESM.docx]

**Supplemental table** *Sensitivity analysis of crude models. Comparing regression coefficients for baseline*

*Use of snuff, smoking and double use with and without baseline adjustments in femoral neck (FN) and total hip (TH) models. The Tromsø Study, Fit Futures.*

| Girls | | | | | | | | | | | | | | | | | |
| --- | --- | --- | --- | --- | --- | --- | --- | --- | --- | --- | --- | --- | --- | --- | --- | --- | --- |
|  |  | *∆*aBMD | | | | | | | | | *∆*BMC | | | | | | |
|  | Baseline adjustment | FN | | TH | | | TB | | | | FN | | TH | | | TB | |
|  |  | β | p | | β | *p* | | β | *p* | β | | *p* | β | *p* | β | | *p* |
| Use of snuff | Unadjusted | -0.015 | 0.006 | | -0.014 | 0.003 | | -0.003 | 0.505 | -0.051 | | 0.017 | -0.373 | 0.027 | -16.492 | | 0.360 |
|  | adjusted | -0.015 | 0.006 | | -0.014 | 0.003 | | -0.003 | 0.311 | -0.073 | | 0.010 | -0.385 | 0.023 | -20.242 | | 0.282 |
| Smoking | unadjusted | -0.011 | 0.175 | | -0.011 | 0.131 | | -0.003 | 0.434 | -0.092 | | 0.026 | -0.342 | 0.176 | 0.977 | | 0.971 |
|  | adjusted | -0.011 | 0.121 | | -0.011 | 0.128 | | -0.004 | 0.322 | -0.096 | | 0.019 | -0.343 | 0.176 | 0.342 | | 0.990 |
| Double use | unadjusted | -0.009 | 0.130 | | -0.006 | 0.234 | | -0.002 | 0.502 | -0.058 | | 0.047 | -0.264 | 0.139 | -7.241 | | 0.704 |
|  | adjusted | -0.009 | 0.123 | | -0.006 | 0.216 | | -0.003 | 0.293 | -0.066 | | 0.024 | -0.276 | 0.122 | 2.794 | | 0.881 |

| Boys | | | | | | | | | | | | | | | | | | | |
| --- | --- | --- | --- | --- | --- | --- | --- | --- | --- | --- | --- | --- | --- | --- | --- | --- | --- | --- | --- |
| *∆*aBMD | | | | | | | | | | | | *∆*BMC | | | | | | | |
|  | Baseline adjustment | FN | | | TH | | | | TB | | | FN | | | TH | | | TB | |
|  |  | | β | p | | β | *p* | β | | *p* | β | | *p* | β | | *p* | β | | *p* |
| Use of snuff | Unadjusted | | -0.020 | 0.012 | | -0.012 | 0.006 | -0.018 | | <0.001 | -0.151 | | 0.003 | -0.933 | | 0.003 | -80.298 | | 0.001 |
|  | adjusted | | -0.019 | 0.008 | | -0.018 | 0.006 | -0.016 | | 0.000 | -0.146 | | 0.004 | -1.004 | | 0.001 | -76.564 | | 0.001 |
| Smoking | unadjusted | | **-0.020** | **0.046** | | -0.022 | 0.008 | -0.019 | | 0.001 | -0.174 | | 0.010 | -1.146 | | 0.005 | -55.659 | | 0.068 |
|  | adjusted | | **-0.009** | **0.398** | | -0.021 | 0.016 | -0.016 | | 0.006 | -0.165 | | 0.014 | -1.127 | | 0.004 | -49.356 | | 0.108 |
| Double use | unadjusted | | -0.019 | 0.025 | | -0.023 | 0.001 | -0.018 | | <0.001 | -0.150 | | 0.010 | -1.209 | | 0.001 | -64.798 | | 0.013 |
|  | adjusted | | -0.021 | 0.017 | | -0.024 | 0.001 | -0.018 | | <0.001 | -0.161 | | 0.005 | -1.127 | | 0.004 | -64.944 | | 0.013 |

*aBMD =Areal bone mineral density (g/cm^2^), BMC = Bone mineral content (g), FN = Femoral neck, TH = Total hip, TB=Total body. Disagreement between models in bold*
